# Supplementation with medium-chain fatty acids increases body weight loss during very low-calorie ketogenic diet: a retrospective analysis in a real-life setting

**DOI:** 10.1186/s12967-023-03880-7

**Published:** 2023-01-16

**Authors:** Claudia Vetrani, Ludovica Verde, Silvia Savastano, Annamaria Colao, Giovanna Muscogiuri, Luigi Barrea

**Affiliations:** 1 Dipartimento Di Scienze Umanistiche, Centro Direzionale, Università Telematica Pegaso, Via Porzio, Isola F2, 80143 Naples, Italy; 2 Centro Italiano per la cura e il Benessere del paziente con Obesità (C.I.B.O), University of Naples “Federico II”, Via Sergio Pansini 5, 80131 Naples, Italy; 3 Department of Clinical Medicine and Surgery, Endocrinology Unit, University of Naples “Federico II”, Via Sergio Pansini 5, 80131 Naples, Italy; 4 UNESCO Chair “Education for Health and Sustainable Development”, University of Naples “Federico II”, Naples, Italy

**Keywords:** Obesity, Nutritional ketosis, VLCKD, Ketogenic diet, Ketone bodies, Medium-chain fatty acids (MCTs), Inflammation, Diet

## Abstract

**Background:**

Very low-calorie ketogenic diet (VLCKD) has shown to significantly reduce body weight and fat mass, as well as inflammation. These effects are supported by nutritional ketosis, which triggers the utilization of the ketone body as an energy source. Medium-chain fatty acids (MCTs) might serve as potential enhancers of ketone bodies production with a greater effect on weight loss. Nevertheless, no clinical studies have evaluated the effect of MCTs supplementation in addition to VLCKD. Therefore, the present study aimed to evaluate whether the supplementation with MCTs can induce a greater weight reduction during the ketogenic phase of VLCKD.

**Methods:**

In this retrospective study, 263 women with overweight/obesity (body mass index, BMI: 35.7 ± 5.3 kg/m^2^) aged 37.5 ± 14.2 years followed one of these dietary protocols for 45 days: (a) Control group, 83 participants (31.6%) (VLCKD without MCTs), (b) VLCKD + MCTs group, 86 participants (32.7%) (MCTs supplementation − 20 g/day- during VLCKD starting from the first day of the active phase), (c) VLCKD + earlyMCTs, 94 participants (35.7%) (MCTs supplementation − 20 g/day-starting from 5 days before the beginning of the VLCKD active phase. Anthropometric measures, body composition, and c-reactive protein (CRP) concentrations were collected at the beginning and at the end (45 days) of the VLCKD intervention.

**Results:**

MCTs supplementation significantly decreased body weight, BMI, and waist circumference as compared to the control group, with a greater effect in the VLCKD + earlyMCTs group. A two-fold decrease in fat mass and an increase in muscle mass were observed in the VLCKD + earlyMCTs group as compared to the control group. As for inflammation, hs-CRP concentrations (assessed as absolute percent change) were significantly lower in the VLCKD + MCTs group (*p* = 0.009) and the VLCKD + earlyMCTs group (*p* = 0.011) than in the control group. A logistic regression model showed that VLCKD + earlyMCTs increase the likelihood of improvement of BMI classes (OR: 1.85, 95% CI 1.02–3.36) also after adjusting for the potential confounding factors.

**Conclusion:**

MCTs supplementation (20 g/day) may be a useful tool to enhance the beneficial effect of VLCKD on the reduction of body weight and fat mass. In particular, MCTs supplementation before the beginning of the VLCKD active phase might facilitate ketosis thus contributing to the effectiveness of the nutritional intervention.

## Introduction

Obesity is recognized as a chronic disease that associates with several comorbidities, such as type 2 diabetes mellitus (T2D), hypertension, dyslipidemia, cardiovascular diseases (mainly coronary heart disease and stroke), sleep disturbance, and some cancers [1–4]. These comorbidities—also known as non-communicable diseases (NCDs)—reduce the quality of life and life span and increase public health costs [3, 5].

Although several strategies have been developed to obtain weight loss, the trend of obesity is dramatically increasing, particularly among young adults and middle-income countries [6, 7].

In addition to lifestyle factors such as physical inactivity, smoking, and alcohol intake, also diet has been established as a highly modifiable risk factor for obesity and NCDs [8].

Among dietary approaches, very low-calorie ketogenic diet (VLCKD) has been appointed as one of the most effective interventions for body weight loss [9, 10]. In addition, it has shown to reduce inflammation and insulin resistance which represent two main triggers for the onset of NCDs [11].

VLCKD consists in a multistep protocol with three main stages: active phase, dietary re-education, and maintenance [12]. The active stage is the most important stage of VLCKD since it allows the achievement of 80% of the target weight loss, with a duration ranging 30–45 days depending on the individual response. Rapid weight loss is obtained through a great energy restriction (600–800 kcal/day) and a sharply sustained nutritional ketosis [12].

Nutritional ketosis occurs when carbohydrate intake is < 50 g/day and, because of carbohydrate restriction, it enhances the oxidation of the fatty acids in the adipose tissue for energy purposes [13, 14]. Indeed, acetyl-CoA is the precursor of ketone bodies (acetoacetate, β-hydroxybutyrate, and acetone) which are used as an alternative fuel in various tissues.

Interestingly, only fatty acids with carbon chain lengths ≤ 8 can cross the inner membrane of the mitochondria independent of carnitine palmitoyl transferase I [15]. In this contest, fatty acids C8 (caprylic acid) might have a stronger ketogenic effect compared to C10 (capric acid) and C12 (lauric acid) [16]. Clinical evidence demonstrated that 20 g of C8 produces a significantly stronger ketogenic response than 10 g of C8 [15]. However, Norgren and colleagues [17] reported that to minimize potential side effects, the dose of C8 should be limited to 15–20 g *per* intake. Triglycerides containing medium-chain fatty acids (MCTs) consist in fatty acids with a carbon backbone with 6–12 carbon atoms linked to glycerol [18, 19]. After ingestion with the diet, MCTs are digested by intestinal lipases and absorbed in the gut as triglycerides containing long-chain fatty acids (LCTs, > 12 carbon atoms) [19]. Unlike LCTs, the fatty acids contained in MCTs can bind albumin and skip the formation of chylomicrons. Therefore, MCTs skip the hydrolysis by plasma lipoprotein-lipase and the consequent deposition in adipose tissue [20]. Then, MCTs directly reach the liver where they can be metabolized more quickly by mitochondrial β-oxidation [21]. However, unlike LCTs, MCTs do not require carnitine-mediated transport to enter the mitochondria. Moreover, MCTs, especially C8 and C10, can also be oxidized in peroxisomes, thus representing a more available source of energy than LCTs [21]. Several studies showed that MCTs supplementation increases β-hydroxybutyrate concentrations with a dose-dependent relationship [13, 22–24]. Consequently, MCTs might endorse “nutritional ketosis” during ketogenic diets [24].

Over a faster metabolism and less deposition in adipocytes, MCTs can significantly influence energy balance, favouring body weight loss independently of dietary energy intake [25, 26].

The mechanisms behind this effect are not completely understood possibly due to the high heterogeneity of studies available so far. Some studies have shown that MCTs might increase thermogenesis and, consequently, affect energy expenditure. Furthermore, the replacement of LCTs with MCTs was associated with a greater reduction of adipose tissue in animal models as well as in humans [27, 28]. This effect could be mediated by the specific action of MCTs on a G-protein coupled receptor (GPR84) in the adipose tissue [27]. In addition, MCTs might increase satiety feelings thus limiting food intake while favouring body weight control [28–31]. Indeed, hyperketonaemia can enhance the anorexigenic effect at the hypothalamic level [29, 30]. Furthermore, some studies suggested that MCTs can modulate the secretion of some gastrointestinal hormones involved in hunger/satiety feelings (ghrelin and YY peptide, respectively) [29, 31].

To date, it is unclear whether the use of MCTs might increase the acute ketogenic response. Nevertheless, a 30 day clinical trial reported that the consumption of caprylic acid (C8; 6 g twice a day) increased plasma β-hydroxybutyrate concentration from ~ 0.1 mmol/L to ~ 0.2 mmol/L [32].

To the best of our knowledge, no previous studies investigated the potential effects of MCTs supplementation during VLCKD for a greater reduction of body weight and fat mass in individuals with overweight and obesity.

Against this background, the present study aimed to evaluate whether the supplementation with MCTs can induce a greater weight reduction during the ketogenic phase of VLCKD. For this purpose, we retrospectively investigated the effect of MCTs supplementation in addition to diet *vs* diet alone in a group of individuals with overweight/obesity undergoing a VLCKD. Over the effect on weight loss, we evaluated whether the association between VLCKD and MCTs supplementation may also affect inflammatory status as demonstrated for VLCKD alone.

## Methods

### Study design and setting

This study was conducted in compliance with the Strengthening the Reporting of Observational Studies in Epidemiology (STROBE) statement checklist [33]. Data from consecutive participants undergoing a VLCKD protocol for weight loss at *Centro Italiano per la cura e il Benessere del paziente con Obesità* (C.I.B.O.) of the Federico II University Hospital (Naples, Italy) were retrospectively collected between September 2021 and September 2022. All participants provided written consent after being informed about the study design. The study has been approved by the Local Ethical Committee (n. 50/20). The present analyses included a total of two time points: at baseline and during the VLCKD active stage after 45 days with the collection of anthropometric measures and body composition.

### Participants

We included 263 healthy participants as individuals with diseases, such as T2D, might have different metabolic responses [16]. Individuals eligible for this study presented the following features: women aged 18–69 years, body mass index (BMI) 25.0–50.9 kg/m^2^ at the beginning of the nutritional treatments. We excluded individuals presenting one or more of the following characteristics: (a) new onset physiological or pathological conditions that represent contraindications for VLCKD (i.e., pregnancy/breastfeeding, individuals with diabetes on insulin therapy, liver or kidney insufficiency, etc.) [10], (b) poor adherence to the dietary intervention (negative test for ketonuria or food behaviours not included in the dietary program referred by the participant); (c) individuals needing treatment with anti-obesity drugs or referred to bariatric surgery; (d) use of drugs, supplements or nutraceuticals that affect energy expenditure or weight loss during the intervention.

### Nutritional intervention

Participants included in the present study underwent a VLCKD with the use of replacement meals (New Penta, Cuneo, Italy) following a 3-stages protocol (active phase, re-education, and maintenance) [12]. After the nutritional status assessment, the dietary plan was prescribed by the endocrinologist and planned by a skilled nutritionist. The VLCKD provided a total daily energy intake < 800 kcal, with 13% carbohydrates (< 30 g/day); 43% protein (1.2–1.5 g/kg ideal body weight); 44% lipids (mainly from extra-virgin olive oil). According to the international recommendations, participants were prescribed a multi-vitamin and saline supplement (complex B vitamins, vitamins C and E, potassium, sodium, magnesium, calcium, and omega-3 fatty acids; PentaCal, Penta, s.r.l., Cuneo, Italy), as reported in previous studies [34–36]. In all participants, physical activity (at least 30 min/day aerobic exercise) was assessed using a YES/NO response, as reported in previous studies [37–39].

Participants followed one of these dietary protocols: (a) Control group, VLCKD without MCTs; b) VLCKD + MCTs group, MCTs supplementation (20 g/day) during VLCKD starting from the first day of the active phase; c) VLCKD + earlyMCTs, MCTs supplementation (20 g/day) starting from 5 days before the beginning of the VLCKD active phase.

To reduce potential confounding factors related to the oil composition, all participants in the VLCKD + MCTs and VLCKD + earlyMCTs groups used only 100% MCT oil (Kanso MCToil 100%, for composition details, see: https: //www.kanso.com/en/p/oil-mct-100). MCTs supplementation (20 g/day) provided 163.8 kcal and 18.2 g of fat.

To improve compliance with the recommendations for diet and physical activity, participants were contacted by phone calls by a skilled nutritionist each week. Moreover, the participants were advised to measure blood β-hydroxybutyrate by test strips (Optium Xceed Blood Glucose and Ketone Monitoring System; Abbott Laboratories, Chicago, IL, USA) at fasting in the morning and to notify the results to the nutritionist.

### Assessment of anthropometric measures and body composition

Anthropometric measures were collected by a single skilled nutritionist at each visit between 8 a.m. and 10 a.m. Weight, height, and waist circumference were detected in participants wearing light clothing and no shoes, after an overnight fast, according to standard procedures [40, 41]. Weight and height were used to calculate BMI (kg/m^2^) [42]. BMI was classified according to the WHO criteria [43]: normal weight (18.5–24.9 kg/m^2^); Overweight (25.0–29.9 kg/m^2^); Obesity class I (30.0–34.9 kg/m^2^); Obesity class II (35.0–39.9 kg/m^2^); Obesity class III (≥ 40.0 kg/m^2^). All measurements were taken while the subject was standing upright with the feet together and the arms hanging closely by the sides, with the subject standing and breathing normally, as previously reported [36–38].

Body composition was evaluated by bioelectrical impedance analysis (BIA). BIA was performed by a phase-sensitive BIA system (an 800 A current with a frequency of 50 kHz BIA 101 RJL, Akern Bioresearch, Florence, Italy) [44, 45] with BIATRODES electrodes (Akern Srl; Florence, Italy), according to the standard procedures of the European Society of Parenteral and Enteral Nutrition (ESPEN) [46]. All measurements were performed under strictly standardized conditions by the same certified skilled nutritionist and with the same device to avoid inter-observer and inter-device variability as reported in previous studies [36, 47, 48]. Briefly, the device was routinely checked with resistors and capacitors of known values. Reliability for intraday and interday measurements by the same observer was < 2% for resistance (R), < 2.5% for reactance (Xc), and < 3.3% for R, < 2.8% for Xc, respectively. The coefficients of variation (CVs) of repeated measurements of R and Xc at 50 kHz were determined in 10 females by the same observer: CVs were 1.4% for R and 1.3% for Xc.

### Assessment of C-reactive protein concentrations

In a subgroup of participants (n = 207), information on c-reactive protein (CRP) was retrieved from electronic medical records. During each visit, fasting blood samples were collected in the morning (8.00–10.00 a.m.), and stored at − 80 °C until processing. Serum high-sensitivity (hs) CRP concentrations were analyzed by CardioPhase^®^ (Siemens Healthcare Diagnostics, Marburg, Germany), based on particle-enhanced immunonephelometry. The CV of intra-and interassay was < 7%.

### Statistical analyses

The data distribution was evaluated by Kolmogorov-Smirnov test and variables not normally distributed were normalized by logarithmic transformation. Skewed variables (waist circumference, R, and muscle mass) were back transformed for presentation in tables and figures. Continuous variables were expressed as mean ± standard deviation (SD) whereas categorical variables were reported as numbers and percentages (%). The effect of MCTs supplementation was evaluated as absolute changes (45 days *minus* baseline). Differences between groups were analyzed by analysis of variance (one-way ANOVA) and post hoc analyses for multiple comparisons (Bonferroni). Differences between categorical variables were assessed by χ^2^ (chi-square) test. A logistic regression model was used to estimation of the likelihood of BMI changes of WHO classes with MCTs supplementation. BMI improvement was investigated as a dichotomous variable (yes/no) and no MCTs supplementation was designated as reference for ease of comparability. Estimates of the logistic regression coefficients were reported as odds ratios (OR). The analysis was conducted in six steps: Model 1 not adjusted; Model 2 adjusted for age; Model 3 adjusted for age and body weight at baseline; Model 4: adjusted for age, body weight at baseline, and percentage of fat mass at baseline. A *p* value < 0.05 was considered significant. Statistical analysis was performed according to standard methods using the Statistical Package for Social Sciences software 26.0 (SPSS/PC; SPSS, Chicago, IL, USA).

## Results

A total of 263 participants were included in the analyses, with 83 participants (31.6%) in the control group (VLCKD alone without any integration with MCTs), while 86 participants (32.7%) in the VLCKD + MCTs group, and 94 participants (35.7%) in the VLCKD + earlyMCTs group. All individuals of the three groups were evaluated at baseline and at the 45th day (the end of the active phase of VLCKD). At baseline, the three groups did not differ for demographic and anthropometric features, as well as for body composition (Table 1). Absolute changes after the intervention were reported in Table 2. MCTs supplementation significantly decreased body weight, BMI, and waist circumference as compared to the control group, with a greater effect in the VLCKD + earlyMCTs group (*p* < 0.001). MCTs supplementation significantly also affected body composition (Table 2). A two-fold decrease in fat mass and an increase of muscle mass were observed in the VLCKD + earlyMCTs group as compared to the control group (*p* < 0.001). Fat mass and muscle mass were also different when comparing the two groups with MCTs supplementation. Of interest, from baseline, no participant in the three groups changed their physical activity levels during the 45 days of VLCKD (χ^2^ = 4.22, *p* = 0.121). All dietary interventions significantly decreased the prevalence of higher BMI classes (obesity class III and obesity class III) from baseline to 45 days of VLCKD active phase while lower BMI classes increased (obesity class I, overweight, and normal weight) (Table 2). Although no significant difference was observed (*p* = 0.623), VLCKD + earlyMCTs induced a threefold increase of normal weight participants (n = 6, 6.4%) than the other two dietary interventions (2 participants for both groups) (Table 2).

**Table 1:** Demographic and anthropometric characteristics, and body composition in the three groups at baseline

Parameters	Control group (n = 83, 31.6%)	VLCKD + MCTs (n = 86, 32.7%)	VLCKD + earlyMCTs (n = 94, 35.7%)	*p* for ANOVA
Age (years)	40.1 ± 15.2	36.8 ± 14.1	35.9 ± 13.1	0.130
Physical activity (yes)	32 (38.6%)	21 (22.3%)	33 (38.4%)	χ^2^ = 4.22, *p* = 0.121
Body weight (kg)	92.5 ± 14.9	98.5 ± 16.5	95.7 ± 17.5	0.059
BMI (kg/m^2^)	35.1 ± 5.1	35.9 ± 5.2	36.0 ± 5.5	0.475
25.0–29.9 kg/m^2^	13 (15.7%)	11 (12.8%)	16 (17.0%)	χ^2^ = 0.64, *p* = 0.725
30–34.9 kg/m^2^	31 (37.3%)	31 (36.0%)	23 (24.5%)	χ^2^ = 4.16, *p* = 0.125
35–39.9 kg/m^2^	23 (27.7%)	23 (26.7%)	37 (39.4%)	χ^2^ = 4.14, *p* = 0.126
≥ 40.0 kg/m^2^	16 (19.3%)	21 (24.4%)	18 (19.1%)	χ^2^ = 0.95, *p* = 0.622
Waist circumference (cm)	106.3 ± 13.7	105.8 ± 15.6	102.2 ± 16.4	0.147
R (Ω)	481.6 ± 68.6	467.9 ± 68.1	483.8 ± 80.5	0.296
Xc (Ω)	47.6 ± 9.8	45.9 ± 8.8	45.3 ± 9.4	0.245
FM (%)	41.6 ± 6.4	42.4 ± 7.3	43.0 ± 7.1	0.403
Muscle mass (%)	27.2 ± 4.3	27.8 ± 5.4	26.8 ± 4.8	0.390

Data are expressed as mean ± SD or n (%). One-way ANOVA and post hoc test for multiple comparisons (Bonferroni) and χ^2^ (chi-square) test

*VLCKD* Very low-calorie ketogenic diet, *MCTs* Medium chain fatty acids, *BMI* body mass index, *R* resistance, *Xc* reactance, *FM* fat mass

**Table 2:** Absolute changes in anthropometric characteristics and body composition in the three groups

Parameters	Control group (n = 83, 31.6%)	VLCKD + MCTs (n = 86, 32.7%)	VLCKD + earlyMCTs (n = 94, 35.7%)	*p* for ANOVA
Body weight (kg)	− 4.8 ± 2.64	− 7.2 ± 1.9^a^	− 8.8 ± 2.9^a,b^	** < 0.001**
BMI (kg/m^2^)	− 1.8 ± 0.9	− 2.6 ± 0.6^a^	− 3.3 ± 1.1^a,b^	** < 0.001**
18.5–24.9 kg/m^2^	2 (2.4%)	2 (2.3%)	6 (6.4%)	χ^2^ = 2.66, *p* = 0.263
25.0–29.9 kg/m^2^	23 (27.7%)	25 (29.1%)	24 (25.5%)	χ^2^ = 0.29, *p* = 0.865
30–34.9 kg/m^2^	28 (33.7%)	26 (30.2%)	35 (37.2%)	χ^2^ = 0.98, *p* = 0.611
35–39.9 kg/m^2^	24 (28.9%)	24 (27.9%)	19 (20.2%)	χ^2^ = 2.16, *p* = 0.340
≥ 40.0 kg/m^2^	6 (7.2%)	9 (10.5%)	10 (10.6%)	χ^2^ = 0.73, *p* = 0.693
Waist circumference (cm)	− 4.4 ± 5.7	− 7.3 ± 5.4^a^	− 8.1 ± 4.9^a^	** < 0.001**
R (Ω)	6.1 ± 36.4	8.9 ± 30.7	− 1.1 ± 49.5	0.227
Xc (Ω)	2.8 ± 6.4	3.8 ± 5.4	5.0 ± 6.7	0.059
FM (%)	− 2.5 ± 2.7	− 3.7 ± 2.6^a^	− 5.1 ± 3.8^a,b^	** < 0.001**
Muscle mass (%)	1.1 ± 2.1	1.8 ± 1.9	2.7 ± 2.6^a,b^	** < 0.001**

Data are expressed as mean ± SD

A *p*-value in bold type denotes a significant difference (*p* < 0.05)

*VLCKD* Very low-calorie ketogenic diet, *MCTs* Medium chain fatty acids, *BMI* body mass index, *R* resistance, *Xc* reactance, *FM* fat mass

^a^p < 0.05 *vs* control; one-way ANOVA and post hoc test for multiple comparisons (Bonferroni)

^b^p < 0.05 *vs* VLCKD + MCTs; one-way ANOVA and post hoc test for multiple comparisons (Bonferroni).

**Fig. 1:**
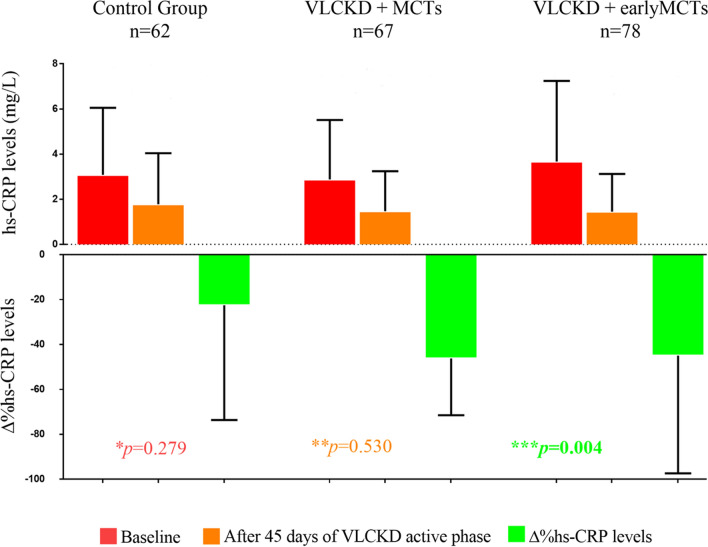
Changes in hs-CRP concentrations in the three study groups. One-way ANOVA and post hoc test for multiple comparisons (Bonferroni). A *p*-value in bold type denotes a significant difference (*p* < 0.05). * hs-CRP concentrations in the three groups at baseline. ** hs-CRP concentrations in the three groups after 45 days of VLCKD active phase. *** The absolute percent change of hs-CRP concentrations in the three groups

Inflammatory status was assessed in a subgroup of 207 subjects, being 62 participants in the control group, 67 participants in the VLCKD + MCTs group, and 78 participants in the VLCKD + early MCTs group. Hs-CRP concentrations did not differ among the three groups at baseline (control group: 3.1 ± 2.9 mg/L, VLCKD + MCTs: 2.9 ± 2.6 mg/L, VLCKD + earlyMCTs: 3.7 ± 3.5 mg/L; *p* = 0.279) as well as at 45 days of VLCKD active phase (control group: 1.8 ± 2.3 mg/L, VLCKD + MCTs: 1.5 ± 1.8 mg/L, VLCKD + earlyMCTs: 1.4 ± 1.7 mg/L; *p* = 0.530) (Fig. 1). However, as compared to the control group, CRP concentrations (evaluated as absolute percent change) were significantly lower in both the VLCKD + MCTs group (∆% = − 22.5 ± 51.2 *vs* − 46.2 ± 25.3; *p* = 0.009), and the VLCKD + earlyMCTs group (∆% = − 22.5 ± 51.2 *vs* − 45.0 ± 52.4; *p* = 0.011) (Fig. 1).Findings from the logistic regression modeling in the whole population were shown in Table 3.

**Table 3:** Logistic regression analyses on the likelihood of BMI improvement * after MCTs supplementation, adjusted for possible confounders

Intervention	Model 1	Model 2	Model 3	Model 4
	OR (95% CI)	OR (95% CI)	OR (95% CI)	OR (95% CI)
Control group (no MCTs)	ref	ref	ref	ref
VLCKD + MCTs	1.53 (0.84–2.77) *p*: 0.131	1.59 (0.87–2.91) *p*: 0.162	1.49 (0.83–2.70) *p*: 0.181	1.48 (0.82–2.67) *p*: 0.190
VLCKD + earlyMCTs	1.98 (1.08–3.63) ***p*: 0.028**	1.96 (1.06–3.60) ***p*: 0.031**	1.90 (1.04–3.48) ***p*: 0.037**	1.85 (1.02–3.36) ***p*: 0.043**

*BMI improvement was evaluated as change of BMI classes according to WHO classification after the intervention. Model 1: unadjusted; Model 2: adjusted for age; Model 3: adjusted for age and body weight at baseline; Model 3: adjusted for age, body weight at baseline, and fat mass at baseline

A *p*-value in bold type denotes a significant difference (*p* < 0.05)

*VLCK* Very low-calorie ketogenic diet, *MCTs* Medium chain fatty acids, *BMI* body mass index, *OR* odds ratios, *CI* confidence interval

MCTs supplementation starting at the beginning of the VLCKD active phase (VLCKD + MCTs) did not influence the likelihood to improve BMI classes. As for VLCKD + earlyMCTs intervention, participants presented a high likelihood of improvement of BMI classes (OR: 1.85, 95% CI 1.02–3.36) also after adjusting for the potential confounding factors (age, body weight at baseline, and fat mass at baseline).

## Discussion

The present study showed that individuals undergoing a VLCKD with daily MCTs supplementation (20 g/day) obtained a higher body weight loss than individuals supplied with VLCKD alone. Weight loss translated into a significant reduction in BMI, waist circumference, and fat mass. These effects were greater when MCTs supplementation started 5 days before the beginning of the VLCKD active phase than on the first day of the dietary protocol.

Our results are in line with previous studies focusing on supplementation with MCTs during energy-restricted diets [25, 26]. Indeed, two meta-analyses [25, 26] showed that the isoenergetic substitution of LCTs with MCTs during energy-restricted dietary interventions resulted in a small reduction in body weight (− 0.5 to − 0.7 kg) and waist circumference (− 1.5 to − 1.8 cm) in middle-aged individuals with overweight/obesity. However, when considering studies involving very low-calorie diets (< 800 kcal/day) with MCTs supplementation the mean weight reduction was similar to that observed in our study (on average − 8 kg).

As reported above, MCTs are metabolised differently from LCTs, since they can reach the liver after being absorbed in the intestine and are largely oxidized and not stored [23]. In addition, MCTs have been suggested to increase thermogenesis and reduce fat deposition, thus contributing to weight loss [27, 28]. Indeed, Hill and colleagues [49] demonstrated that MCTs s increased thermogenesis by 50% after a 6 day supplementation. Therefore, this mechanism might explain the greater effect on body weight loss that we observed in the group starting MCTs supplementation prior to the VLCKD.

As for the effect on body composition, MCTs supplementation significantly reduce fat mass whereas muscle mass was increased only in the earlyMCTs group as compared to VLCKD alone.

It is known that during nutritional ketosis, ketone bodies can be used as the main energy source thus limiting protein breakdown for energy purposes [50]. On the other hand, high doses of MCTs have been shown to stimulate lipolysis by increasing lipoprotein lipase activity in animal models [51, 52]. Nevertheless, the mechanisms underlying the effect of MCTs on body composition in humans need further clarification.

In the management of obesity and its metabolic comorbidities, VLCKD has been proposed also to reduce systemic inflammation by virtue of its antioxidant and anti-inflammatory effects [53, 54]. Interestingly, in our study all groups undergoing the VLCKD presented a reduction of CRP concentrations, a well-known marker of inflammation. This result confirms those obtained in previous studies demonstrating the anti-inflammatory effects of the VLCKD in the short [36, 54] and -long-term in individuals with obesity [55]. Indeed, VLCKD has shown to reduce inflammation through several mechanisms, i.e.by inhibiting activation of the nuclear factor kappa-light-chain-enhancer of activated B cells, and the inflammatory nucleotide-binding, leucine-rich-containing family, pyrin domain-containing-3, and inhibiting histone deacetylases [56]. Notably, the earlyMCTs group experienced the greatest reduction as compared to the other groups, likely due to the overflow of ketone bodies [57]. Unfortunately, we did not perform a quantitative measurement of ketone bodies and we were not able to test this hypothesis.

Our study has some strengths and limitations. To the best of our knowledge, this is the first study evaluating the effect of MCTs supplementation in addition to VLCKD in individuals with overweight/obesity. In addition, this study was performed in a large population in a real-life setting.

Weaknesses included the single-centre recruitment with potential selection bias. Nevertheless, to increase the homogeneity of the study population, we included only women to avoid potential gender differences in body composition and CRP concentrations. In addition, we did not evaluate the long-term effect of MCTs supplementation. However, the short study duration increased participants’ compliance to the treatment. Finally, we did not analyse other inflammatory markers, but CRP is a reliable inflammatory biomarker in different clinical settings [58].

Another limitation might be the transferability of these results to other populations. Our study focused on young adult women with overweight/obesity. Previous studies reported that MCTs supplementation did not increase ketone bodies in middle-aged and elderly subjects [59]. Therefore, further studies of the ketogenic effect of MCTs in different populations are warranted.

## Conclusion

**Fig. 2:**
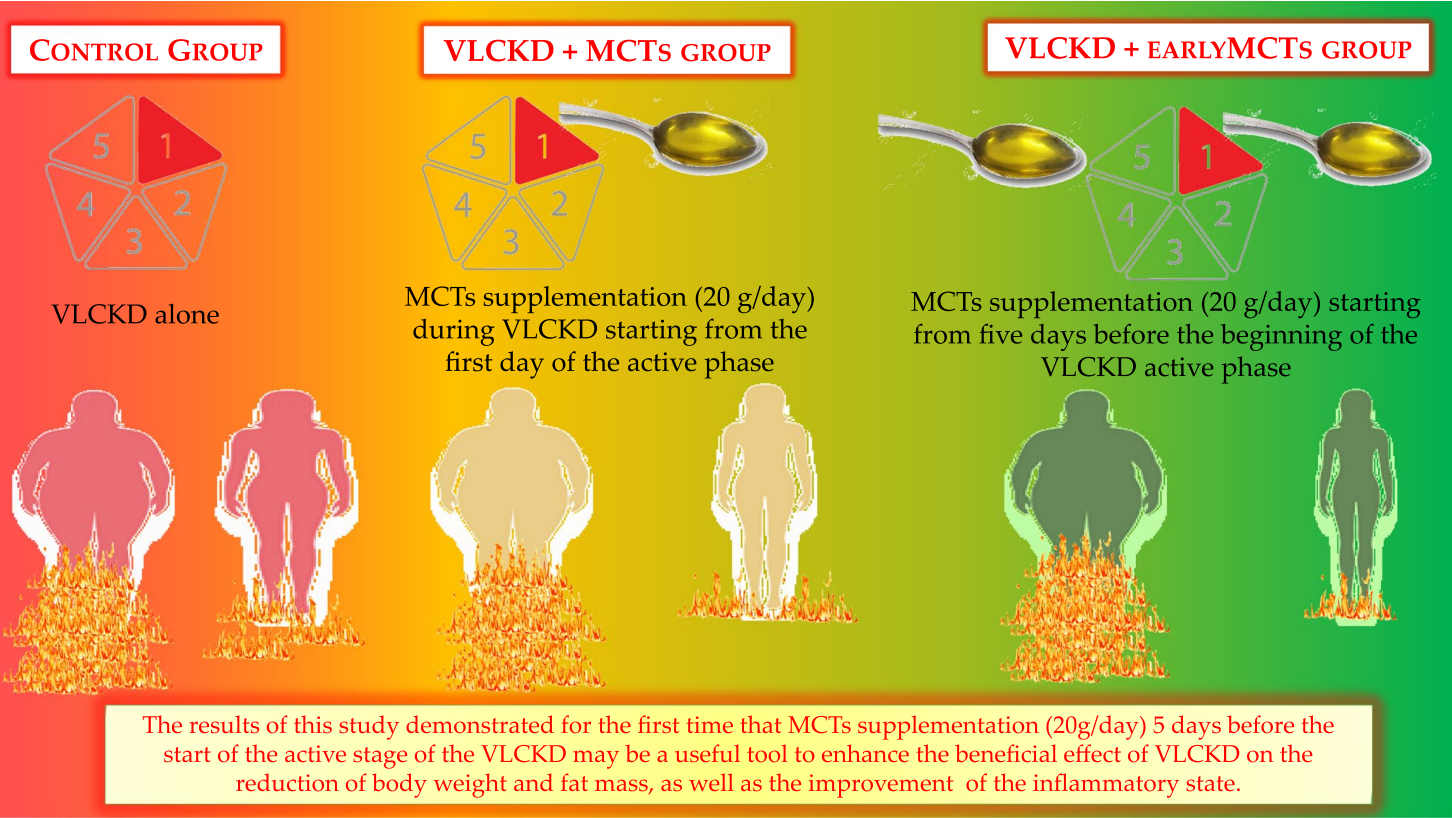
MCTs supplementation during the active stage of the VLCKD. The MCTs supplementation (20 g/day) 5 days before the beginning of the VLCKD active phase might facilitate the transition into ketosis thus contributing to the effectiveness of the nutritional intervention enhancing its beneficial effects on weight loss, body composition modulation and inflammatory status

The results of the present study demonstrated for the first time that MCTs supplementation (20 g/day) during the active stage of the VLCKD may be a useful tool to enhance the beneficial effect of VLCKD on the reduction of body weight and fat mass, as well as the improvement of the inflammatory state. In particular, MCTs supplementation 5 days before the beginning of the VLCKD active phase might facilitate the transition into ketosis thus contributing to the effectiveness of the nutritional intervention and enhancing its beneficial effects (Fig. 2). However, further studies extending the observations to subsequent stages of the VLCKD are mandatory. In addition, VLCKD with early MCTs supplementation (5 days before the onset of the active phase) should be compared with other hypocaloric dietary programs to confirm its role in the enhancement of weight loss and reduced inflammation by virtue of the increase of ketosis. Finally, this study underlines the pivotal role of the nutritionist in the management and correct planning of the VLCKD.

## Data Availability

Not applicable.

## Abbreviations

BMI: Body mass index
CI: Confidence interval
CRP: C-reactive protein
LCTs: Long-chain fatty acids
MCTs: Medium-chain fatty acids
OR: Odds ratio
T2D: Type 2 diabetes mellitus
VLCKD: Very low-calorie ketogenic diet
